# Evaluation of a new e-learning framework for teaching nuclear medicine and radiology to undergraduate medical students

**DOI:** 10.1177/2058460119860231

**Published:** 2019-07-18

**Authors:** Ankush Gulati, Thomas Schwarzlmüller, Elsa du Plessis, Eirik Søfteland, Robert Gray, Martin Biermann

**Affiliations:** 1 Nuclear Medicine/PET Centre, Department of Radiology, Haukeland University Hospital, Bergen, Norway; 2 Section for Radiology, Department of Clinical Medicine, University of Bergen, Bergen, Norway; 3 Department of Clinical Science, University of Bergen, Bergen, Norway; 4 Section for Anesthesiology, Department of Clinical Medicine, University of Bergen, Bergen, Norway; 5 Department of Anaesthesia and Intensive Care, Haukeland University Hospital, Bergen, Norway; 6 Section for Pedagogy, Department of Psychology, University of Bergen, Bergen, Norway

**Keywords:** Computed tomography, CT, positron emission tomography, PET, education

## Abstract

**Background:**

Radiology and nuclear medicine have traditionally been taught in a series of lectures and seminars concluded by an examination testing knowledge rather than skills.

**Purpose:**

To emphasize skills in the students’ learning process, we developed and evaluated a new e-learning framework for teaching medical imaging.

**Material and Methods:**

The framework consists of electronic lectures, a learning management system (LMS), and a diagnostic viewing system. Students were to review positron emission tomography/computed tomography (PET/CT) examinations of five cases of lung cancer. The framework was evaluated in an objective structured clinical examination (OSCE) taken by 139 undergraduate students at the end of their third year, and in a comparative survey of two groups of 85 and 84 students in the fifth and sixth year who were taught the same oncology course with and without mandatory PET/CT exercises, respectively.

**Results:**

Of the 139 third-year students, 134 (96%) passed the OSCE PET/CT station according to the predefined criteria. While 85/85 (100%) of the fifth-year students had taken exercises when they were mandatory, only 2/84 (2%) of the sixth-year students had reviewed the cases on a voluntary basis (*P* < 0.001). Of the 25 survey responders in the fifth year, 24 (96%) agreed that the mandatory PET/CT exercises had promoted their learning while the sixth-year students, whose course concluded with a multiple-choice examination, emphasized the utility of online lectures.

**Conclusion:**

The new e-learning framework teaches students basic competency in PET/CT navigation and interpretation and is associated with a high degree of student satisfaction.

## Introduction

Traditionally, radiology and nuclear medicine have been taught in series of lectures supplanted by teaching in smaller student groups based on case presentations. Little emphasis was placed on teaching students competencies such as reading and interpreting relevant radiological or nuclear medicine studies on their own. A typical course would be concluded by an oral examination or, when academic staff was short, by a multiple choice (MCQ) examination.

The introduction of a new medical curriculum at the University of Bergen in 2015 prompted us to take a fresh approach to teaching medical imaging (1,2). Inspired by Dee Fink’s theory on “significant learning” (3), we decided to use our e-learning framework to teach third-year medical students how to read and interpret PET/CT, even before they had been taught CT navigation in their radiology course. Opting for depth rather than coverage, we focused on a single, epidemiologically relevant disease—lung cancer—and created an e-learning tutorial that taught students how to use diagnostic medical imaging software to read PET/CT studies of five cases with different stages of lung cancer.

We evaluated our new framework for teaching nuclear medicine in two contexts: (i) an objective structured clinical examination (OSCE) with 146 third-year students; and (ii) a direct comparison of 85 and 84 students taught the same nuclear medicine/oncology module at the end of the fifth and sixth year, but with different post-teaching activities (read five PET/CTs versus MCQ examination) under the new and old medical curriculum.

## Material and Methods

### E-learning framework

**Fig. 1.**
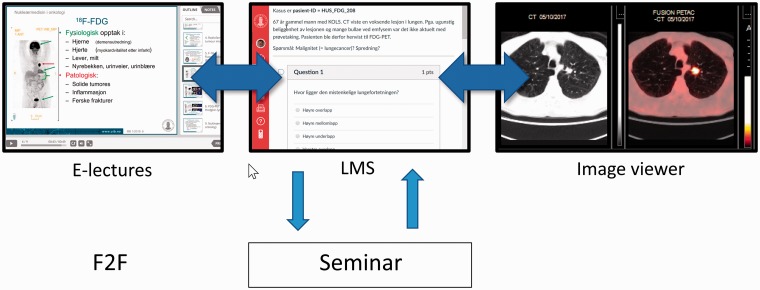
E-learning framework for teaching nuclear medicine and radiology. The e-learning framework consists of electronic lectures (e-lectures; https://www.uib.no/radionett/nuklear), a learning management system (LMS; https://mitt.uib.no) and diagnostic image viewing system. It can be extended by face-to-face (F2F) teaching as an optional component depending on the target audience.

Our new e-learning framework consists of electronic lectures (e-lectures) available on the Internet (https://www.uib.no/radionett/nuklear), an e-learning system (also called learning management system [LMS]), and a diagnostic viewing system for nuclear medicine including PET/CT (Fig. 1) (1,4). This framework can be extended by face-to-face (F2F) teaching, such as seminars, if required by the curriculum (2).

### OSCE

OSCE was introduced at the University of Bergen in June 2018 at the end of the third year of medical school. Stated briefly, an OSCE consists of a set number of “stations” in which a student has to solve a predefined medical problem within a set time period (e.g. 8 min) against a predefined “objective” standard, i.e. a standardized checklist with explicit criteria. Several circuits comprising identical stations but with different examiners and patient simulators are set up in parallel. Within each circuit, students will rotate until they have passed through all stations (5). Traditionally, stations will cover a clinical problem such as taking a patient’s blood pressure or performing a rectal examination on a phantom. We argued that modern medicine includes competencies performed on a digital patient. Our PET/CT station was based on a diagnostic PET/CT viewing program showing the [^18^fluor]-deoxy glucose (FDG) PET/CT of a 67-year-old patient with a 13-mm lesion in the left lung. Students had to find the lung lesion, classify it as FDG-positive or negative, conclude upon malignancy, find metastatic spread, and recommend the most appropriate therapy (curative or palliative) (Supplementary Materials 1). Assessment criteria were clarified in a consensus meeting between all nuclear medicine examiners two weeks before the OSCE. The OSCE station on PET/CT, including the preparatory e-learning materials, had been developed and tested in an OSCE pilot with 40 volunteer students in November 2017. The first compulsory OSCE, which included the new PET/CT station, was conducted in June 2018 with 146 students at the end of their third year.

### OSCE preparation

To prepare students for the PET/CT OSCE station, we created an e-learning tutorial based on our previously published architecture (1). An MCQ quiz was programmed in the university’s LMS (Canvas; https://www.canvaslms.com), which was to be run against specified cases in our teaching case database (Oasis 1.9.4.9; Segami Corp., Inc.; Maryland/MD). To answer the quiz questions, students would have to open the data set using the Oasis diagnostic imaging viewer on a workstation in the hospital network. The quiz gives students instant formative feedback. E.g. when a student reports too small a diameter of the index lung tumour, the student is advised to switch the CT view to a lung window rather than the soft tissue window. When the basic tutorial (“level 1”) is passed, the LMS unlocks level 2 with five cases of lung cancer in different stages. For each of the five cases, the same standardized set of seven MCQ questions have to be answered simulating a typical clinical workflow (Supplementary Materials 2). Level 3 of the quiz covers the same five cases, but in random order.

### “Nuclear medicine in oncology” (NMiO)

NMiO was a new module to be taught to 10th-term students under the new curriculum starting in March 2018. We revised our previous course material from the old curriculum (2). We created new Internet lectures, each < 10 min and on a specific topic. Before participating in the compulsory 90-min seminar, students had to answer a short MCQ examination on three mandatory web lectures (FDG-PET, DOTATOC-PET, and PSMA-PET). The seminar was conducted as a series of interactive case presentations, usually by a volunteer student, projecting original diagnostic images on two large screens in combination with an electronic classroom response system (2). The same new module NMiO was taught in the 10th and the 12th term by the same instructor (M.B.) using identical teaching materials with each group consisting of 40 students. However, activities after F2F teaching differed: under the new curriculum, students did not have any formal MCQ examination, but had to perform the e-learning material originally developed for OSCE in the third year as a mandatory learning activity. Under the old curriculum, students were offered e-learning material on the LMS to prepare for a 30-min MCQ exam in November 2018. The MCQ exam was run on an independent system and included 20 questions, 7 in text and 13 in image format. Students in the 12th term had access to the same practical exercises as students in the 10th term, but on a voluntary basis. All our courses were complemented by online discussion forums in which students could post questions and comments which would be answered within 24 h.

### Student survey

An anonymous student survey was conducted using our previously published methodology (1). The survey was opened to students who had completed the compulsory exercise in the 10th term and to students in the 12th term directly after the completing the electronic MCQ exam. Students in both terms were alerted to the survey by a single announcement in the LMS. The survey closed on 30 November 2018. The survey was approved by the local data protection officer.

### Statistics

To compare proportions of students responding, Fisher’s exact test was used under R (6). The Kruskal–Wallis test was used for between-group comparisons of Likert scales as well as for comparison of the time spent on course work in the 10th and 12th term. *P* values < 0.05 (two-sided) were assumed to indicate statistical significance.

## Results

### OSCE in third-year students

Of 39 students who participated in the OSCE pilot in November 2017, 34 (87%) passed the PET/CT station with a mean score of 86 ± 17% (mean ± standard deviation), while five failed. Of the 139 students who took the PET/CT OSCE station at the end of the third year in June 2018, 134 (96%) passed the PET/CT station with a score of 89 ± 13% (mean ± standard deviation). Five students failed. Because of a temporary network outage, seven students out of a total of 146 OSCE attendees could not take the PET/CT station in the allotted time frame and the PET/CT station was annulled from the final OSCE character.

### Survey in fifth- and sixth-year students

The survey comparing teaching under the new and old curriculum was answered by 26/85 students (31%) in the fifth year (10th term) and 15/84 students (18%) in the sixth year (12th term) (*P* = 0.07, not significant; n.s.). The detailed survey results including the free text responses are available as Supplementary Material 3.

Fifth-year students unanimously agreed (25 fully, one partially) that practical exercises using diagnostic medical imaging software had promoted their learning (Fig. 1) while only one of the sixth-year students (7%; *P* < 0.001) uttered the same preference. While 84/84 (100%) passed the PET/CT tutorial including the five lung cancer cases, which were mandatory, only 9/84 (11%) of the 12th-term students took the PET/CT tutorial and only two (2%) read the five lung cancer cases (*P* < 0.001) as voluntary learning activities.

Students in the 10th term reported a median 2.0 h (interquartile range = 2.0–3.0) working with the compulsory PET/CT exercise, while 12th-term students claimed a median of 2.8 h (2.4–3.3) to prepare for the MCQ examination (n.s.).

## Discussion

We present a new flexible e-learning framework for teaching students medical imaging consisting of e-lectures, LMS, and a diagnostic image viewing system (Fig. 1). While e-learning has been applied to teaching nuclear medicine before (7), the implementation based on the Moodle LMS did not include an image viewing system.

We evaluated our e-learning framework twofold: (i) by testing learning outcomes in an OSCE examination with 139 students at the end of the third year; and (ii) by comparing two independent groups of 85 and 84 students in their fifth and sixth year taught under two different curricula. While the fifth-year students utilized the new framework including mandatory case review in a diagnostic image viewing system, the sixth-year students were instructed using our previous, more traditional e-learning approach without mandatory case review.

Results from the first compulsory OSCE examination in third-year students document that >95% of all students exposed to our new teaching framework competently performed basic image navigation and interpretation using diagnostic PET/CT viewing software. These results compare favorably with results of an OSCE pilot in radiology in second-year medical students at the University of Utrecht in 2004 (8).

**Fig. 2.**
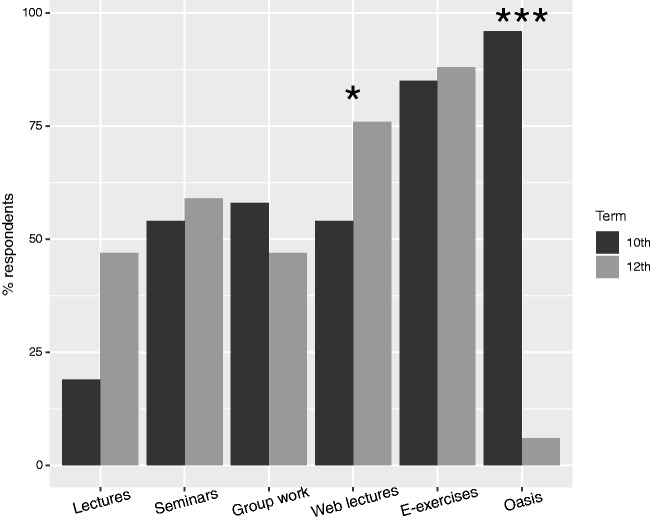
Student survey on activities that promote learning. The plot shows the proportion of students who responded “Agree” when asked if a given learning aid or activity promotes their learning. ***P < 0.001, *P < 0.05 Kruskal–Wallis test comparing the item’s Likert scores between the two student groups.

Our survey of students at the end of their fifth and sixth years documents a marked change in attitude in students exposed to the new framework: 24/25 fifth-year students, who had to review five lung cancer cases using diagnostic PET/CT viewing software, valued using the diagnostic imaging software on authentic cases as the single most important learning activity, while only 1/15 responders in the sixth year, who had to pass the MCQ examination, realized the relevance of using a diagnostic image viewer for her learning (Fig. 2). Even though all students in both years were given access to the same teaching cases inside the LMS, only 2/84 sixth-year students (2%) performed the review of the five lung cancer cases. Our explanation is that the mode of examination steered learning behavior. Since the mandatory MCQ examination emphasized reproduction of knowledge rather than practical skills in image-based problem solving, even the most advanced students chose what we deem to be inferior learning strategies of memorization rather than training image-based clinical problem-solving.

It has been surmised that the beneficial effect of blended learning, i.e. combining F2F instruction with e-learning, is largely due to the increased time students spend with the learning materials (9). It was therefore surprising to find that 12th-term students reported spending a similar amount of time on preparation for the MCQ examination as it took 10th-term students to perform on the mandatory PET/CT exercises.

Using authentic teaching materials to solve real-life problems has been promoted to achieve lasting, significant learning outcomes (3). Qualitative research in undergraduate medical students taught radiology has shown important differences in cognition when students interpret two-dimensional (2D) images such as conventional X-ray or planar scintigraphy versus when they learn how to navigate and interpret three-dimensional (3D) datasets such as CT, MR, and PET (10,11). Interpretation of 2D images focuses most on the discrimination of normal from abnormal findings while interpretation of 3D datasets requires more comprehensive human-computer interaction such as scrolling up and down or adjusting window settings. Den Boer et al. conclude that 3D datasets should be used for teaching radiology. A systematic review by the same group found 11 original articles evaluating e-learning programs for teaching radiology to undergraduate medical students (12). In seven studies, the e-learning program incorporated visualization of stacks of images such as a CT study. Only in 2/7 studies, students were additionally given the possibility to adjust contrast settings in the image stacks. Navigating 3D nuclear medicine hybrid datasets such as PET/CT involves, however, two extra degrees of freedom: adjusting the contrast settings in the nuclear medicine dataset independently of the CT dataset; and overlaying nuclear medicine and CT signals with a variable blending factor (alpha-blending). Rather than trying to integrate a nuclear medicine volume viewer into an e-learning program, we chose to employ our routine clinical nuclear imaging software with a dedicated teaching database containing only anonymized image datasets. This enhances the experience of authenticity for the students and trains the appropriate cognitive domains while keeping the e-learning architecture lean and modular.

In our new e-learning framework for medical imaging consisting of e-lectures, LMS, and image presentation program, any component can be exchanged independently of the others. In our recent student course on endocrine surgery (https://www.uib.no/en/course/ELMED318), the image presentation component for teaching thyroid cytology was a microscope in combination with original cytology slides. In the context of teaching radiology, most modern Picture Archiving and Communication Systems (PACS) allow the generation of anonymized teaching files; however, few PACS will contain diagnostic nuclear medicine viewers, at least not without expensive add-on licenses.

A further important aspect of our e-learning framework is that it saves teaching staff time once the learning materials are implemented. While we chose to arrange F2F seminars with hands-on instruction when launching the PET/CT station in the OSCE pilot in November 2017, students essentially taught themselves before the OSCE in 2018 using the e-learning material that had been developed for the OSCE pilot. Since all teaching materials within the new framework are designed as small modules, they can easily be reused when teaching other groups. In the Netherlands, the e-learning platform developed for undergraduate student education was extended to encompass specialist training in radiology (13). Likewise, materials developed for undergraduate medical students will form the core of our new e-courses under the new system of medical specialist training in Norway (1).

This study had some limitations. First, the completion of the task in the Canvas LMS in fifth-year students does not indicate if the task had been performed by an individual or a small group of students. Indeed, we encouraged group work to enhance learning. However, >80% of the students preparing for OSCE in the third year had similarly taken the exercise in small groups and demonstrated consistently good performance on an individual level. Second, we did not test practical image reading skills in students in the 12th term. However, our results from the OSCE pilot in 2017, in which students took part on a voluntary basis with varying degree of preparation, indicate that it is not possible to pass the PET/CT station without adequate practical training. Finally, the response rate in our survey among the 12th-term students was low, but in line with previous non-compulsory surveys (2).

In conclusion, an e-learning framework consisting of e-lectures, a LMS, and compulsory e-learning exercises involving reading and interpreting a CT or PET/CT examination using diagnostic medical image viewing software is an effective tool for teaching students fundamental competencies in radiology and nuclear medicine, and is associated with a high degree of student satisfaction.

## Supplemental Material

Supplemental material for Evaluation of a new e-learning framework for teaching nuclear medicine and radiology to undergraduate medical students Click here for additional data file. Supplemental Material for Evaluation of a new e-learning framework for teaching nuclear medicine and radiology to undergraduate medical students by Ankush Gulati, Thomas Schwarzlmüller, Elsa du Plessis, Eirik Søfteland, Robert Gray Jr Martin Biermann in Acta Radiologica Open

## Data accessibility

Supplementary data files 1 (PET/CT OSCE station), 2 (PET/CT OSCE preparation) and 3 (Student survey responses) are available from the SAGE author gateway.
