# Supplementary material for: Evaluation of a new e-learning framework for teaching nuclear medicine and radiology to undergraduate medical students
Source: Acta Radiol Open. 2019 Jul 18;8(7):2058460119860231. doi: 10.1177/2058460119860231 (PMC6640061; doi:10.1177/2058460119860231)
Supplement: Supplemental material for Evaluation of a new e-learning framework for teaching nuclear medicine and radiology to undergraduate medical students [file Supplemental_Material.pdf]

Supplementary materials 1: PET/CT OSCE station

The following is the translation of the paper form containing the description of the PET/CT OSCE station at the University of Bergen in June 2018.

OSCE Station owner

Martin Biermann, Nuclear Medicine/PET-center

Required equipment

- Oasis compatible hospital laptop computer
- Wired LAN access
- The examiner is responsible for setting up the laptop including establishing access to the hospital network via her personal smartcard.
- Table and two chairs

Written instruction to the patient

Not applicable

Written instruction to the student

(To be read by the student while she waits for a maximum of two minutes outside the station.)

You are physician in the Lung department at Haukeland University Hospital. You are preparing yourself for the next multidisciplinary lung meeting by looking at the PET/CT study of a patient with a lung lesion. Look at the examination and demonstrate the findings.

66-year-old male patient with chronic obstructive lung disease. A computed tomography because of persistent cough had shown a newly developed lung lesion. Because the lesion was in a difficult location for primary biopsy, the patient was referred to a PET/CT examination with radioactive glucose (F-18-FDG).

You will find the PET/CT examination already loaded into the diagnostic image viewing software. Find the lung lesion. Determine its diameter in millimetres. Evaluate if the lesion has tracer uptake. Measure the intensity of the uptake with the image viewing program. What is the most likely diagnosis? Does the PET/CT examination give an indication of disease stage? If yes, show the relevant findings. What treatment do you suggest?

N. B. You are not required to remember all questions under the examination; the examiner will ask you the pertinent questions and help you move along.

Student name

Evaluation checklist

		Fail<br>0 pt.	Partial<br>1 pt.	Pass<br>2 pts.
1	The student demonstrates the lung lesion on the screen using a CT lung window			
2	The student correctly measures the greatest axial diameter in millimetres			
3	The student gives a correct visual assessment of the lesional FDG uptake ("intense), more than in the mediastinum)			
4	The student measures the correct SUV_{max}			
5	The student concludes that the lesion is malignant			
6	The student shows metastatic spread to and ipsilateral hilar lymph node			
7	The student can show the lymph node in a soft tissue window			
8	The student sees that there are no distant metastases			
9	The student suggests curative treatment (operation or stereotactic radiation therapy depending on the patient's lung function)			
	Sum of points (maximum 18)			

Examiner’s comments (free text):

Rating scale

Examiner’s impression of the student’s performance (tick only one box):

**Failed**	**Borderline**	**Satisfactory**	**Good**	**Excellent**

Date, examiner’s signature

Supplementary materials 2: OSCE preparation

*The following is a translation of the e-learning task on case # ***_FDG_208 (same case as in the actual OSCE examination) in the university's e-learning system (https://mitt.uib.no; https://canvaslms.com).*

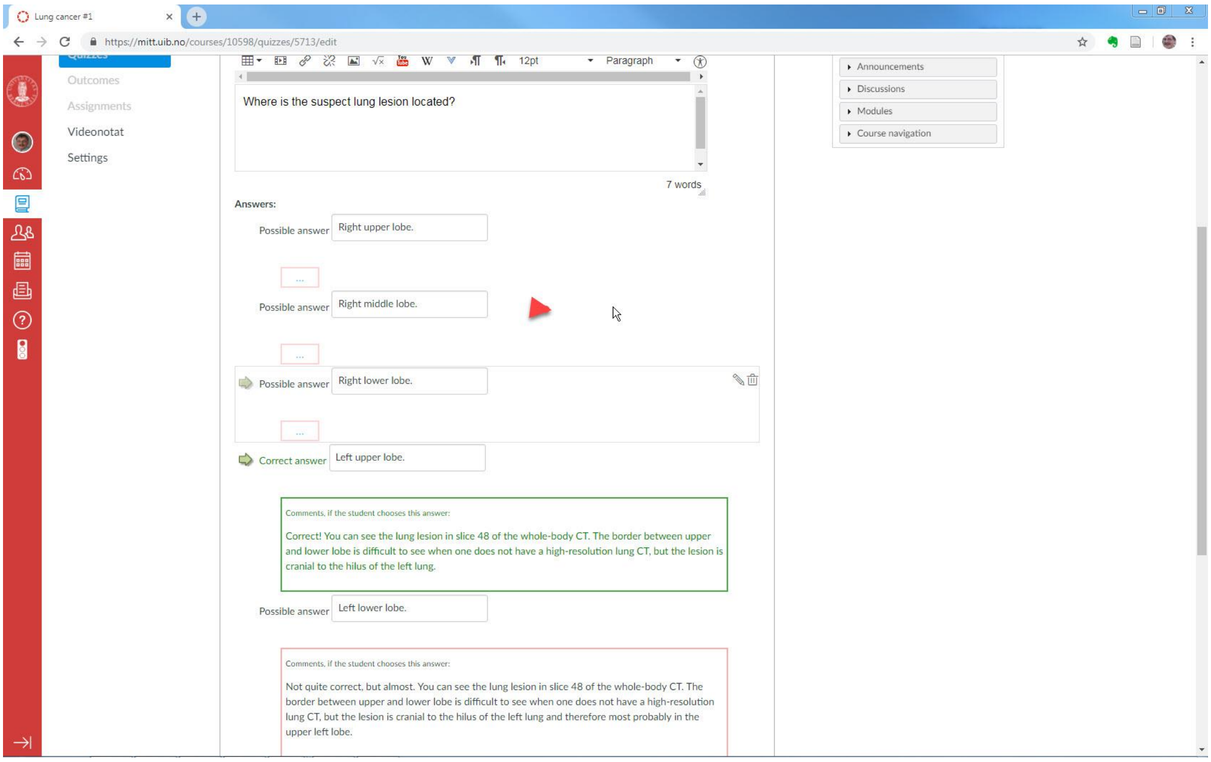

Fig. 1: Screenshot of activity in the Canvas learning management system in editing mode.

Each question relates to a single patient who was examined at the Lung department at Haukeland University Hospital and who underwent PET/CT. Every case will have the same 7 questions!

The current case is **patient ID = ***_FDG_208**

67-year-old male with COPD. CT showed a progressive lesion in the lung. Because of the localization of the lesion and lung emphysema, no biopsy was taken. The patient was therefore referred to FDG-PET.

Question 1: Where is the suspect lung lesion located?

Answer alternatives (only 1 correct):

- Right upper lobe.
- Right middle lobe

- Right lower lobe
- * Left upper lobe (correct)
  Item feedback (green):
  Correct! You can see the lung lesion in slice 48 of the whole-body CT. The border between upper and lower lobe is difficult to see when one does not have a high-resolution lung CT, but the lesion is cranial to the hilus of the left lung.
- Left lower lobe
  Item feedback (red):
  Not quite correct, but almost. You can see the lung lesion in slice 48 of the whole-body CT. The border between upper and lower lobe is difficult to see when one does not have a high-resolution lung CT, but the lesion is cranial to the hilus of the left lung and therefore most probably in the upper left lobe.

Question feedback (red): In case you found something in the right lung: Are you looking at the correct patient? Check that the patient ID in Oasis (which is listed as the first item in line 2 of the header) is the same as the patient ID in the introduction to this case. -
Looking up wrong patient is a classic blunder! It happens to me about twice a year, and it usually takes med som 5 - 10 minutes before I find out.

Question 2: How big is the lung lesion in millimetres?

Please measure the maximum axial diameter in mm. Enter whole numbers only (no decimal separator).

Correct answers between 12.0 and 16.0.
Question feedback:
I measure approx. 13 mm in axial section 48. There are several possibilities why your answer could be wrong:
- You need to select the lung window to obtain the correct diameter. When you measure in soft tissue window, the diameter will appear smaller than the true measurement.
- Have you chosen the slice with the largest axial diameter? The correct slice is #48 of the whole-body CT series.
- Did you mix up cm and mm?

Question 3: When you look at the PET images, does the lesion have FDG-uptake?

Answer alternatives (only 1 correct):
- No
- Slight
- *Intense (correct)

Question feedback: Compare uptake in the lung lesion with physiological soft tissue uptake in the mediastinum, which is considered "weak". Compared with the mediastinal uptake, the uptake of the lung lesion is “intense”.

Question 4: Measure the lesion's FDG uptake as maximum standard uptake value (SUV_{max}). Type whole numbers without decimal separator.

Correct Answers - Between 9.0 and 10.0

Question feedback:

SUV_{max} is a fairly robust parameter that is observer-independent. In routine clinical practice, we use SUV_{max}, not SUV_{mean}. The SUV_{mean} requires that one draws a precise volume of interest (VOI) around the lesion, which is time-consuming and a potential error source. SUV_{max} does not require a precise VOI. Therefore, we use SUV_{max} rather than SUV_{mean}. Possible sources of error:

- Have you looked at the right focus?
- Have you read the correct numbers in Oasis? You must read the red number after "Max".

Question 5: Given the FDG uptake of the lesion, what is the most likely diagnosis?

Answer alternatives (multiple true/false; several may be correct):

- Benign nodule
- Highly differentiated lung tumor (e.g. neuroendocrine tumours).
- *Lung cancer (Correct)

Question feedback:

Intense FDG uptake in a growing lesion strongly suggests cancer.

Question 6: Do you see signs of metastatic spread on the FDG PET/CT?

Answer alternatives (multiple true/false; several may be correct):

- No spread
- *Ipsilateral lung hilus (correct)
  - Item feedback: On CT-slice 58 of the whole-body CT, focal FDG uptake is seen with SUV-max 3.9 in a lymph node in the hilus of the left lung. Use the normal soft tissue window (S = soft tissue) to find it.
- Contralateral lung hilus
- Mediastinum
- Distant metastases

Question 7: What treatment would you suggest for this patient?

Answer alternatives (multiple true/false; several may be correct):

- Only follow-up, no treatment
- *Surgery (curative) (correct)
- *Radiation Therapy (curative) (correct)
- Palliative treatment

Question feedback:
This how the patient’s case unfolded:
A growing FDG-positive lung lesion suggested lung cancer. Surgical excision was performed. The final diagnosis was non-small cell lung cancer in the left upper lobe with two lymph node metastases in the left hilus. We take the histological findings as confirmation that our PET diagnosis was correct. That final histology reveals more lymph node metastases than seen on imaging is not unusual.

Supplementary Materials 3: Student survey responses

The user survey was conducted in Norwegian using the Moodle “Feedback” activity under Moodle 3.5. The following is a transcript of the raw survey results. Graphs were created using “R” after export of the complete survey data in comma separated value format. To test for difference between student groups, Fisher’s exact test was used for proportions (number of respondence), and Kruskal-Wallis test for Likert scales (question 1 ff.) and for the time spent on the learning tasks (question 4), making no assumptions of a normal distribution. A p-value < 0.05 (two-sided) was assumed for statistical significance.

The authors’ comments on selected survey results or responses are typed in *italics*.

Informed consent

All respondents (41 of 41) gave their informed consent to participate in the survey.

Respondents

In the 10th term, 26 out of 85 students (31 %) responded, in the 12th term 15 out of 84 (18 %) (p = 0.07, n. s. Fisher’s exact test).

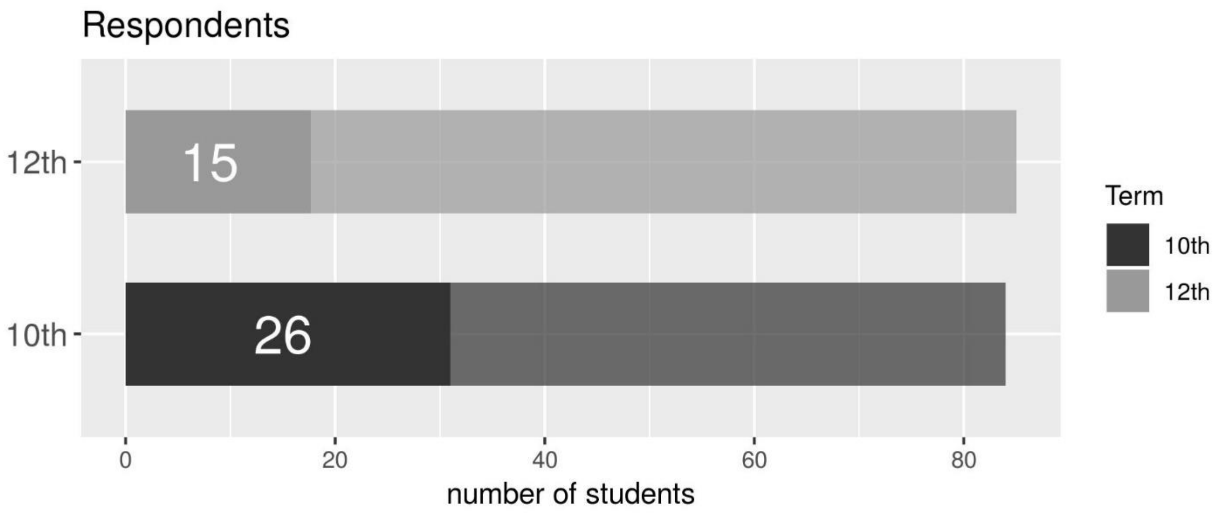

Authors’ comment: The higher proportion of respondents in the 10th term may suggest a higher level of student engagement. However, students in the 12th term underwent their main examinations in internal medicine and surgery at the end of their studies immediately after finishing the course in radiology and nuclear medicine.

Questions 1 to 4 focused the main course activity and thus differed between 10th term and 12th term students.

Question 1: Relevance of MCQ (12th term)/compulsory activity (10th term)

“I perceived the questions in the multiple-choice examination as relevant for my future medical profession.” (12th term) – “I perceived the obligatory learning activity (look at 5 FDG PET/CT examinations in patients with lung cancer) as relevant for my future medical profession.” (10th term)

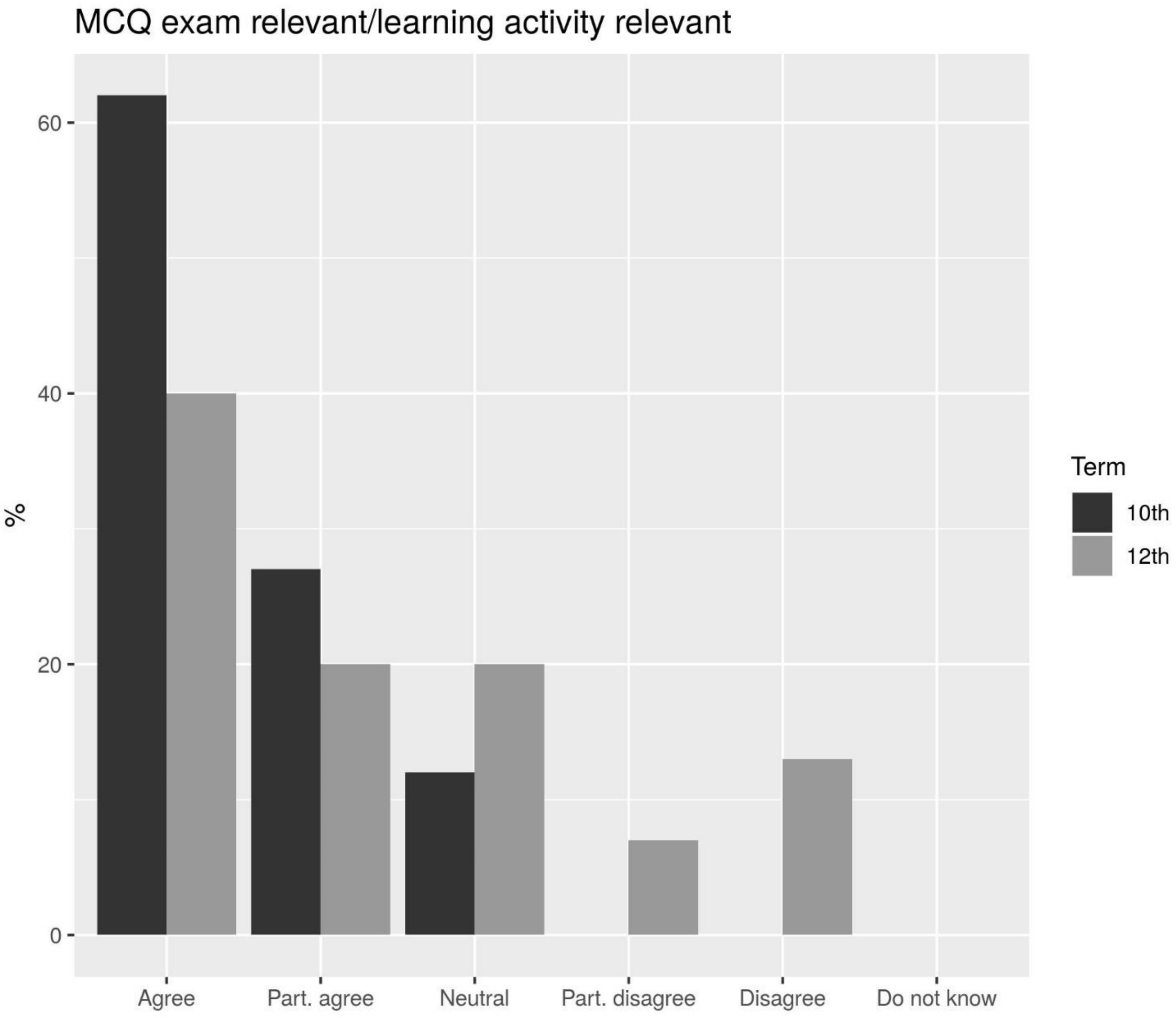

Authors’ comment: A greater proportion of students in the 12th term doubt the relevance of the MCQ examination while a larger proportion of the 10th term students perceives the compulsory learning activity as relevant; however the difference in responses does not meet statistical significance (n.s).

Question 2: Fairness of scoring (12th term)/formative feedback (10th term)

“I perceived the scoring of the multiple-choice examination as fair.” (12th term) – “I perceived the formative feedback from the e-learning system as fair/constructive.” (10th term)

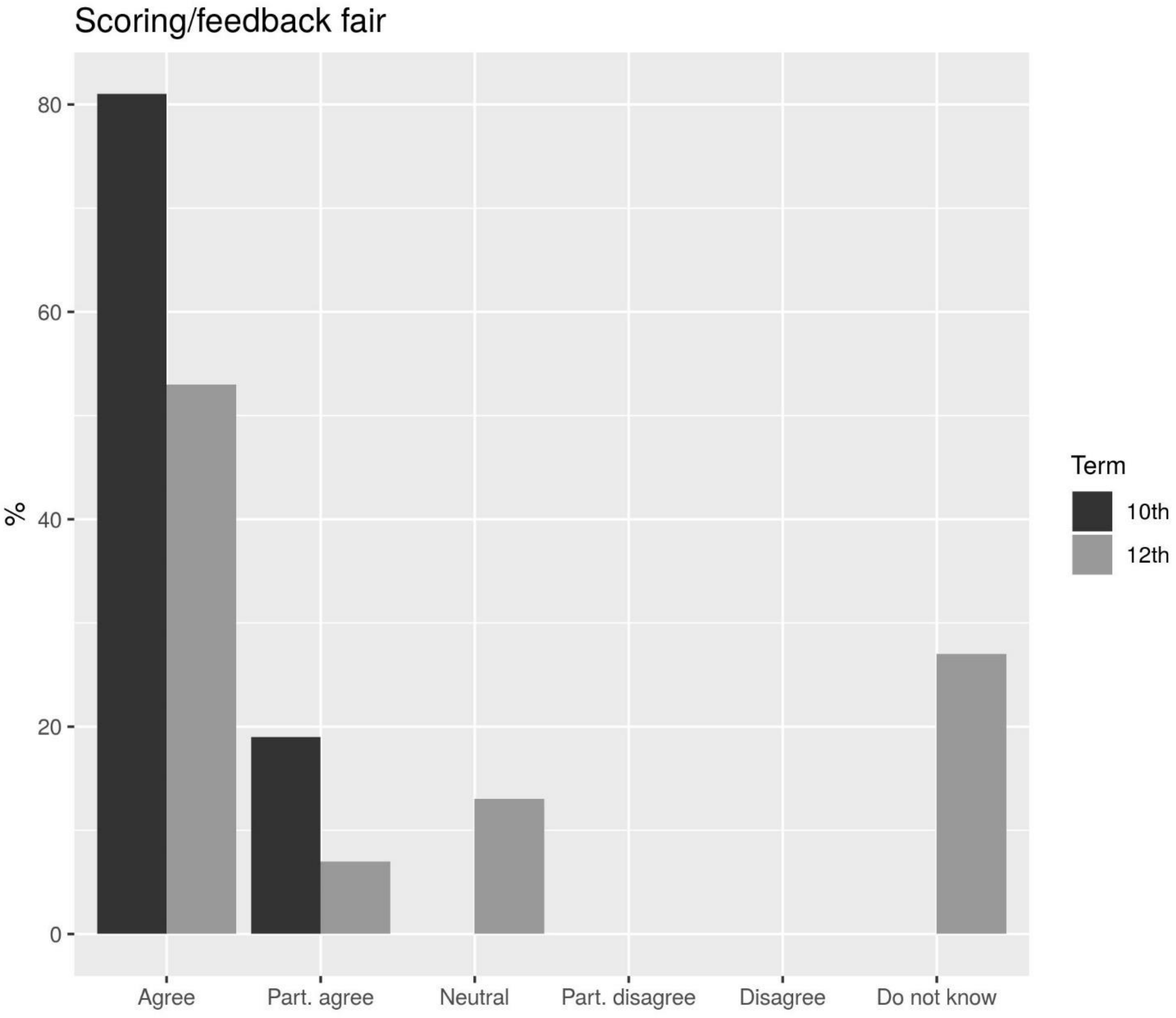

Authors’ comment: The response of “Do not know” of 23 % the 12th term students is correct in that the results of the MCQ-exam were not disclosed. All of the 12th term students passed with “A”, the top grade; only 1 student passed with “B”. The difference between the responses of 10th and 12th term students is statistically significant ($p < 0.05$).

Question 3: Good teaching material

"I was offered good learning aids to prepare myself for the multiple-choice examination." (12th term) – "I was offered good learning aids in order to prepare myself for the compulsory learning activity." (10th term)

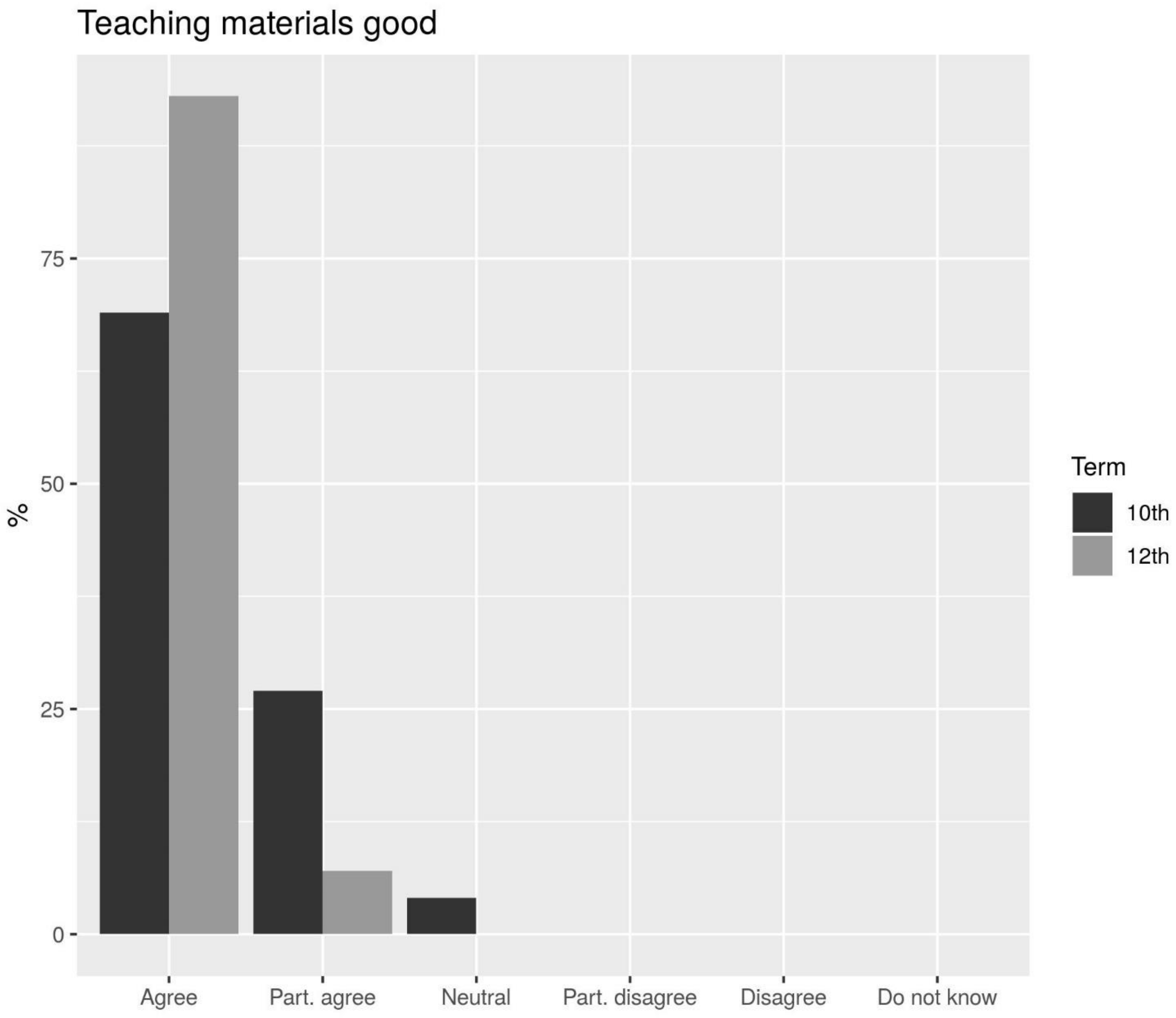

Authors' comment: Students in the 10th term appear less satisfied than students in the 12th term (n.s.). The probable explanation is that there was very close alignment between the MCQ-questions in the teaching material on https://mitt.uib.no and the actual examination on https://vurdering.uib.no. The teaching materials in the 10th term were similar, but they were to provide foundational knowledge and guidance for a practical task, which resided in a different domain.

Question 4: Time spent (in hours)

"How much time (hours) did you spend preparing the multiple-choice exam? How much of your time (in percent) did you use on the oncology part of the multiple-choice examination?" (12th term) – "How much time (in hours) did you spend on doing the obligatory learning activity?" (10th term)

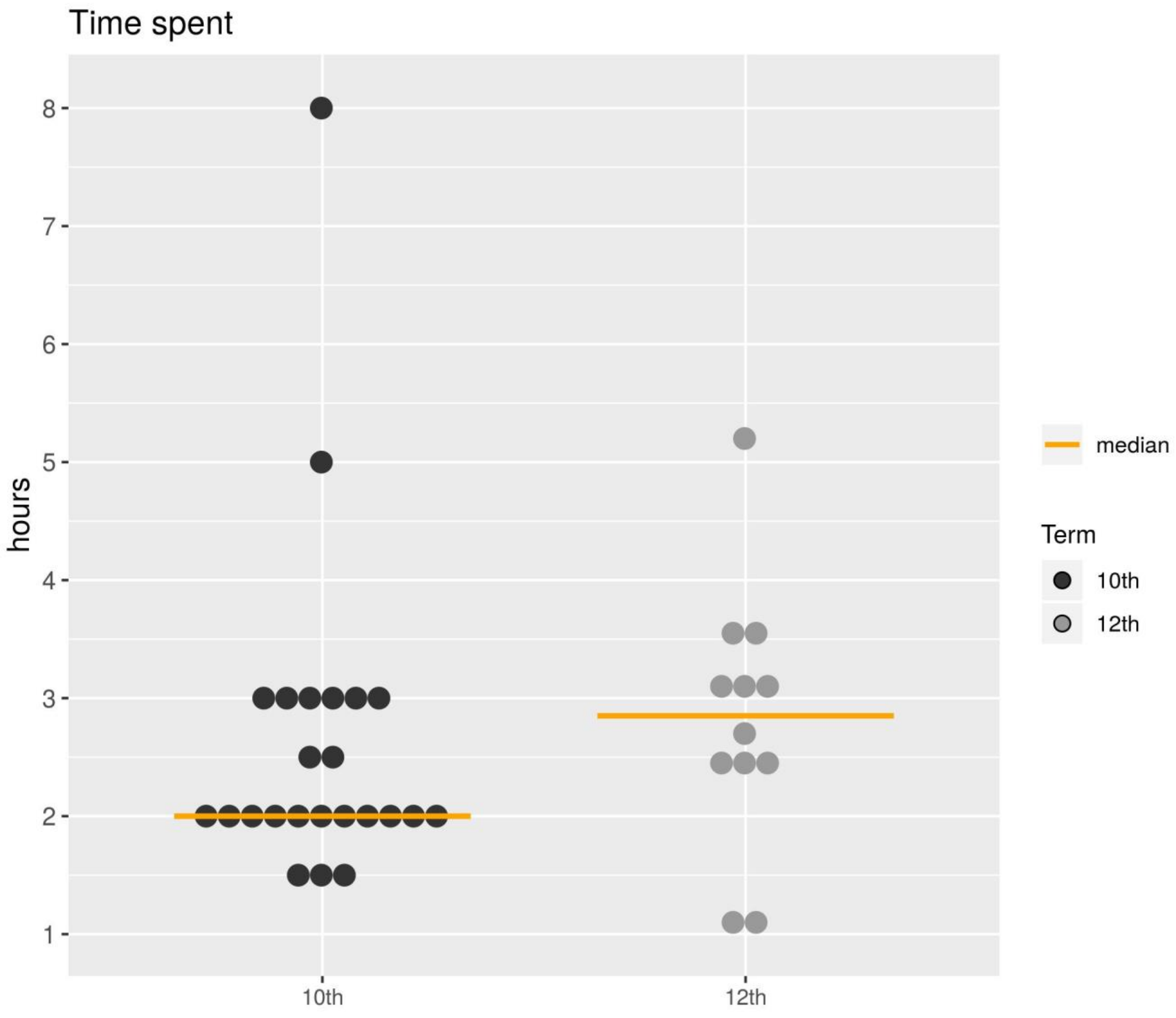

Authors' comment: It is very surprising that the 12th year students report spending more time on preparing the MCQ examination than the 10th year students on the compulsory learning activity (n.s.).

Questions 5 to 14 were identical for all students and assessed students' learning preferences.

Question 5: Plenary lectures

"Plenary lectures promote my learning."

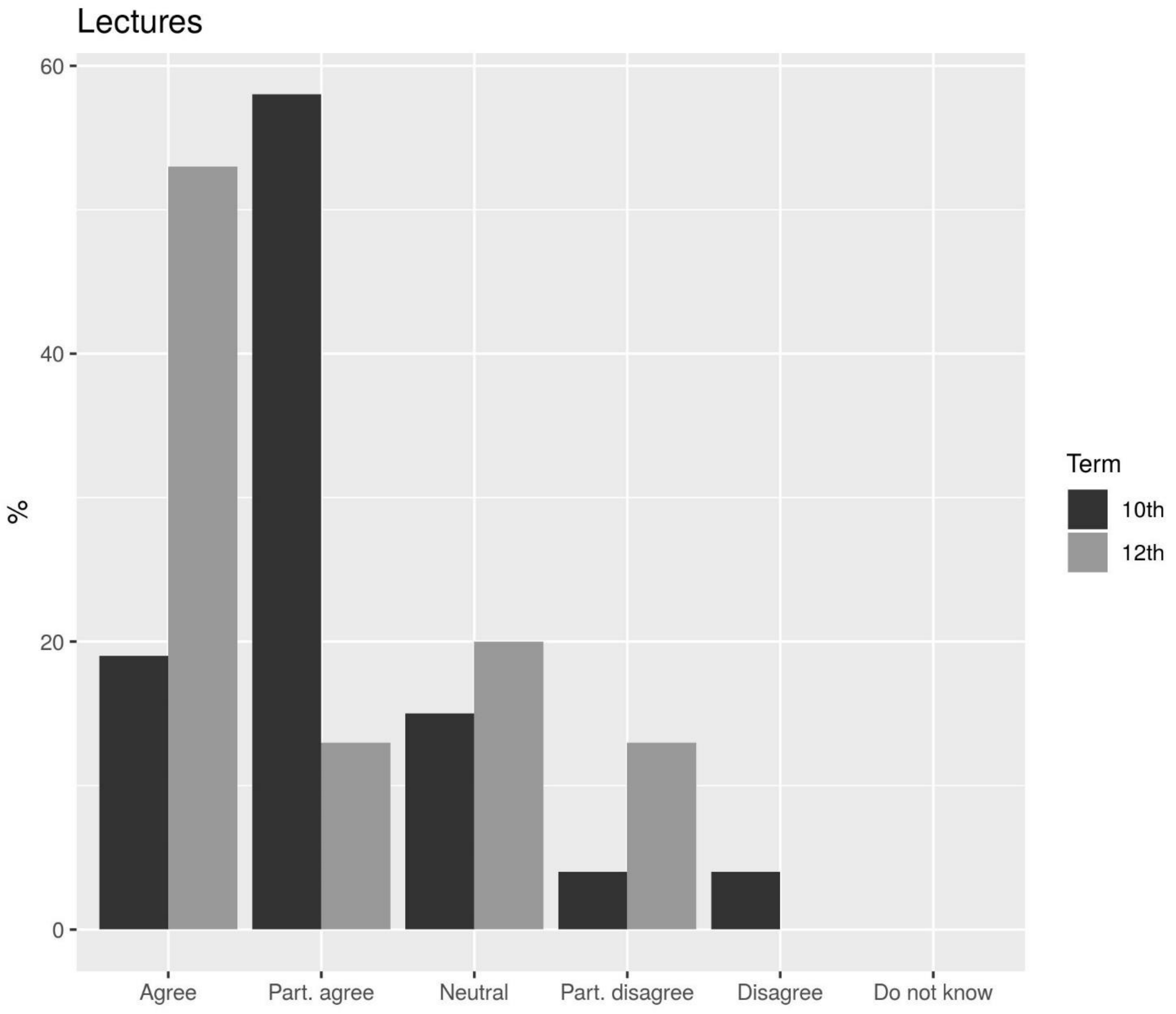

Authors' comment: 12th-term students appear to see greater benefit in plenary lectures than 10th-term students. A possible explanation is that the old curriculum put a greater emphasis on lectures than the new (n.s.).

Question 6: Web lectures

"Web lectures promote my learning."

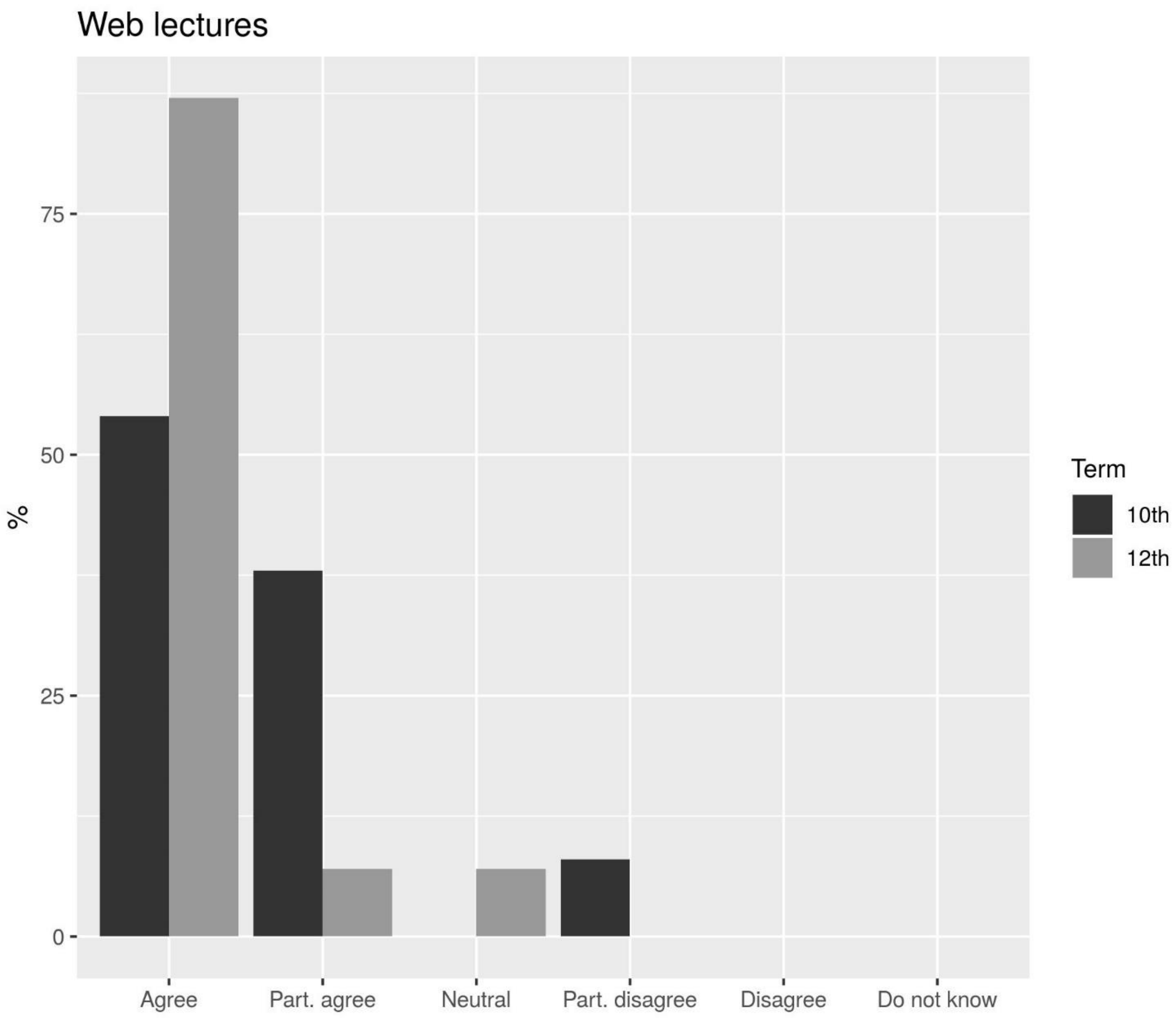

Authors' comment: Web lectures receive the second highest satisfaction rating by 10th-term students in the survey while 12th-term students are less affirmative ($p < 0.05$).

Question 7: Seminars

"Seminars promote my learning."

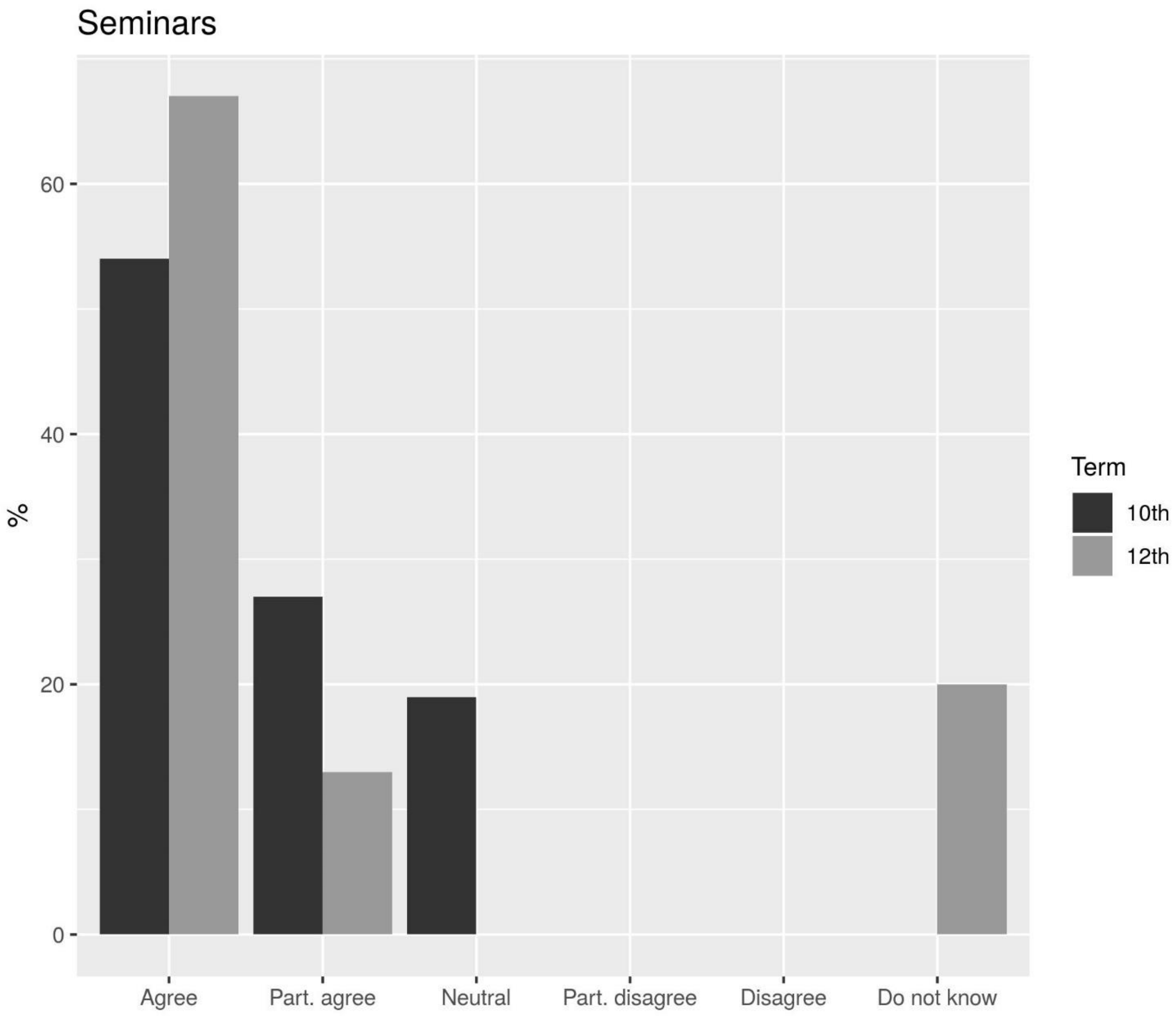

Question 8: Group work

"Group work together with other students promotes my learning."

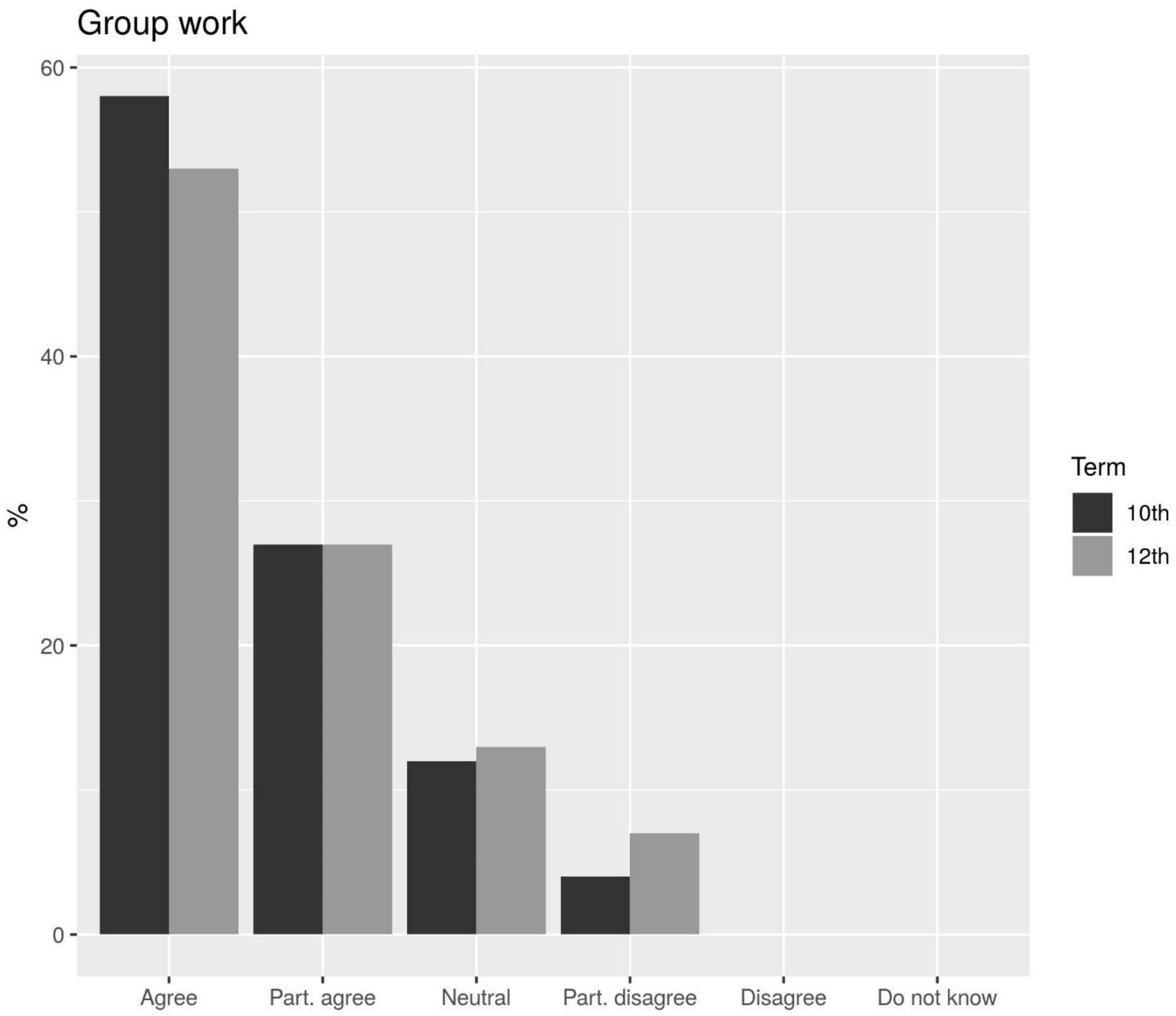

Question 9: E-learning exercises

"Exercises in the e-learning system mitt.uib.no promote my learning in medical imaging."

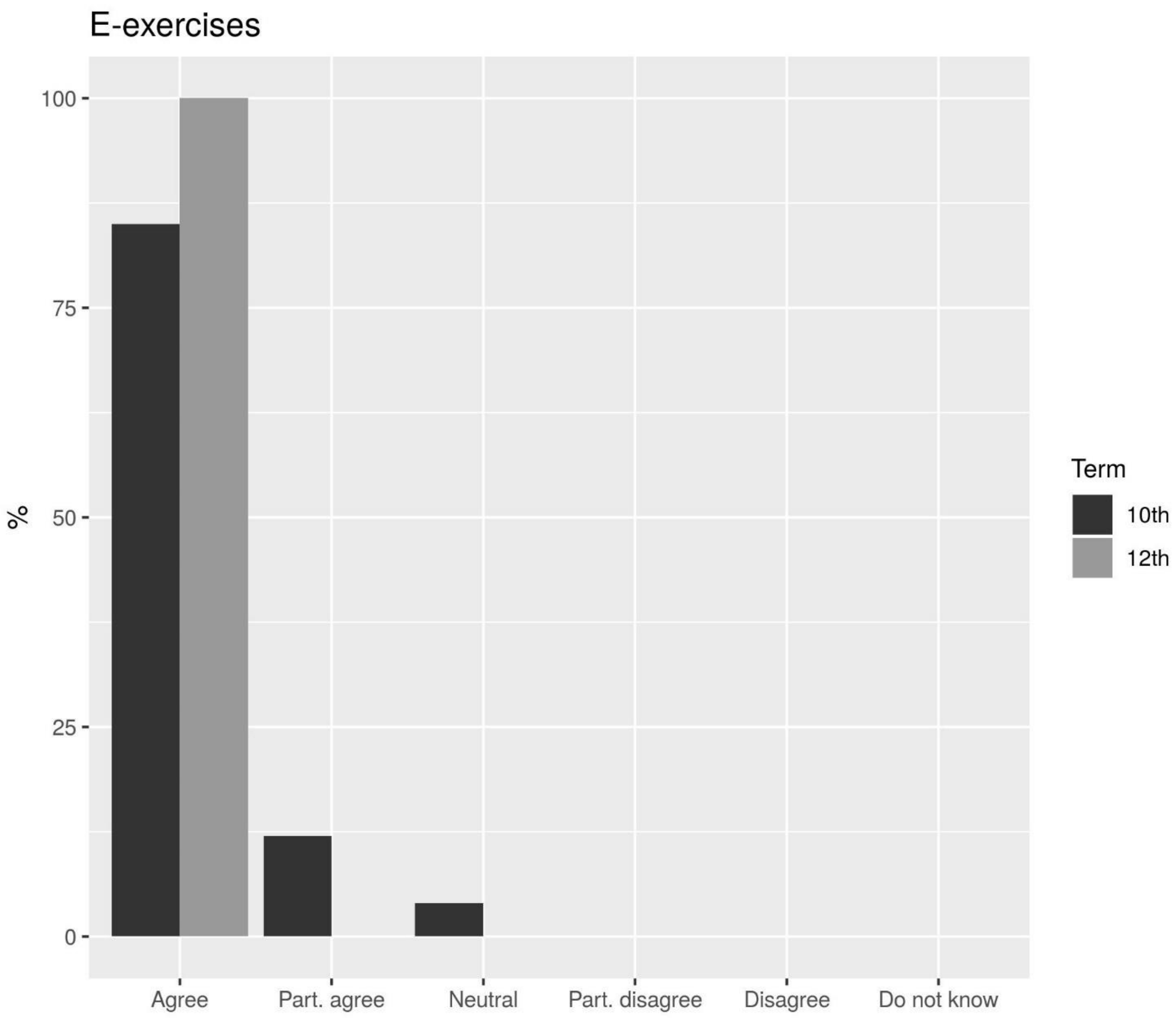

Question 10: Medical imaging software

"Working with a medical imaging program such as Oasis promotes my learning in medical imaging."

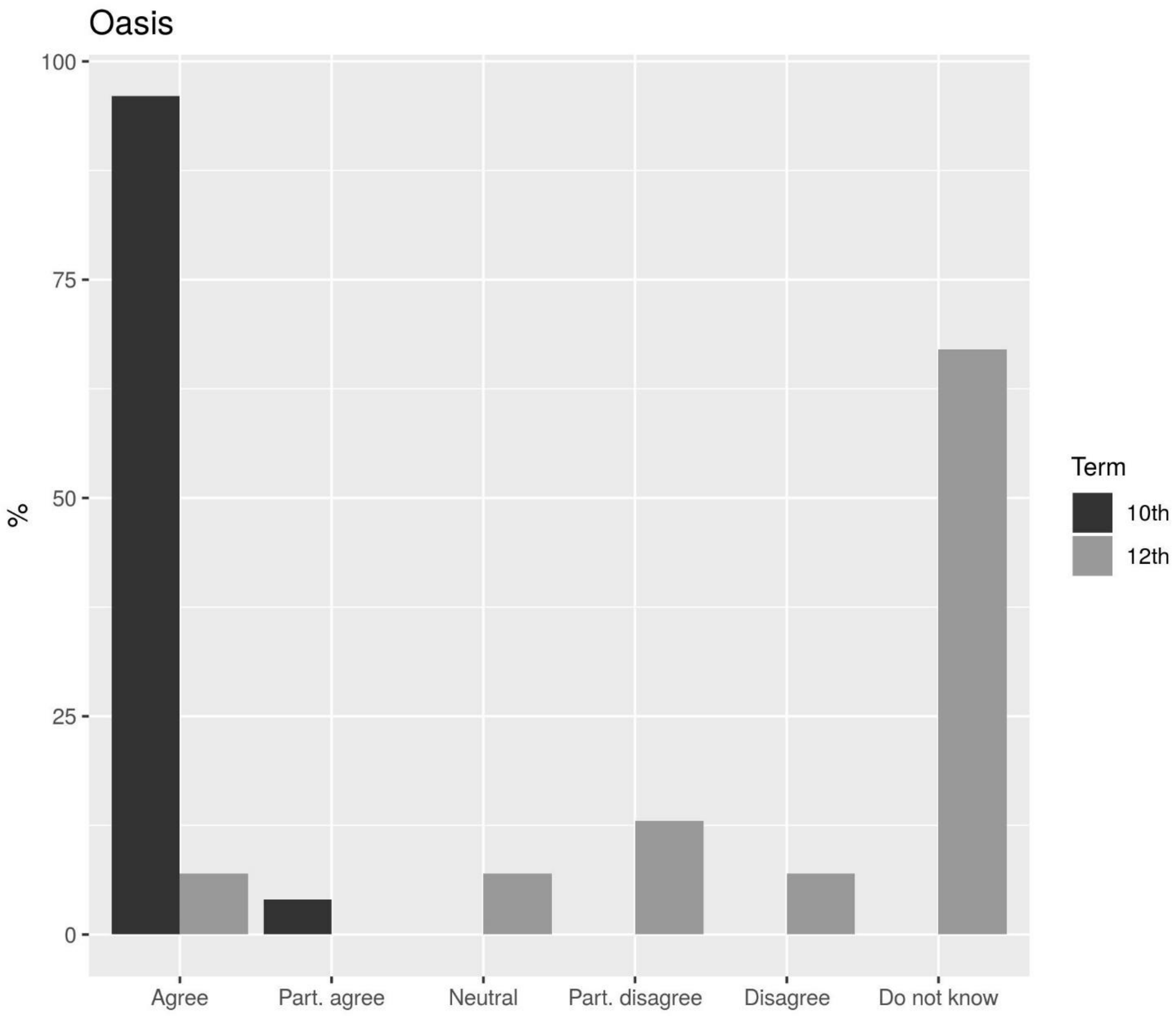

Authors' comment: Students in the 10th term give the use of dedicated medical image viewing software the highest satisfaction rating in the survey (p < 0.001). The proportion of 12th term students who respond in the negative without ever having experienced this mode of teaching is surprising but illustrates the deficiencies of the old curriculum, which was largely lecture-based.

Question 11: Oral examinations

"Oral examinations promote my learning."

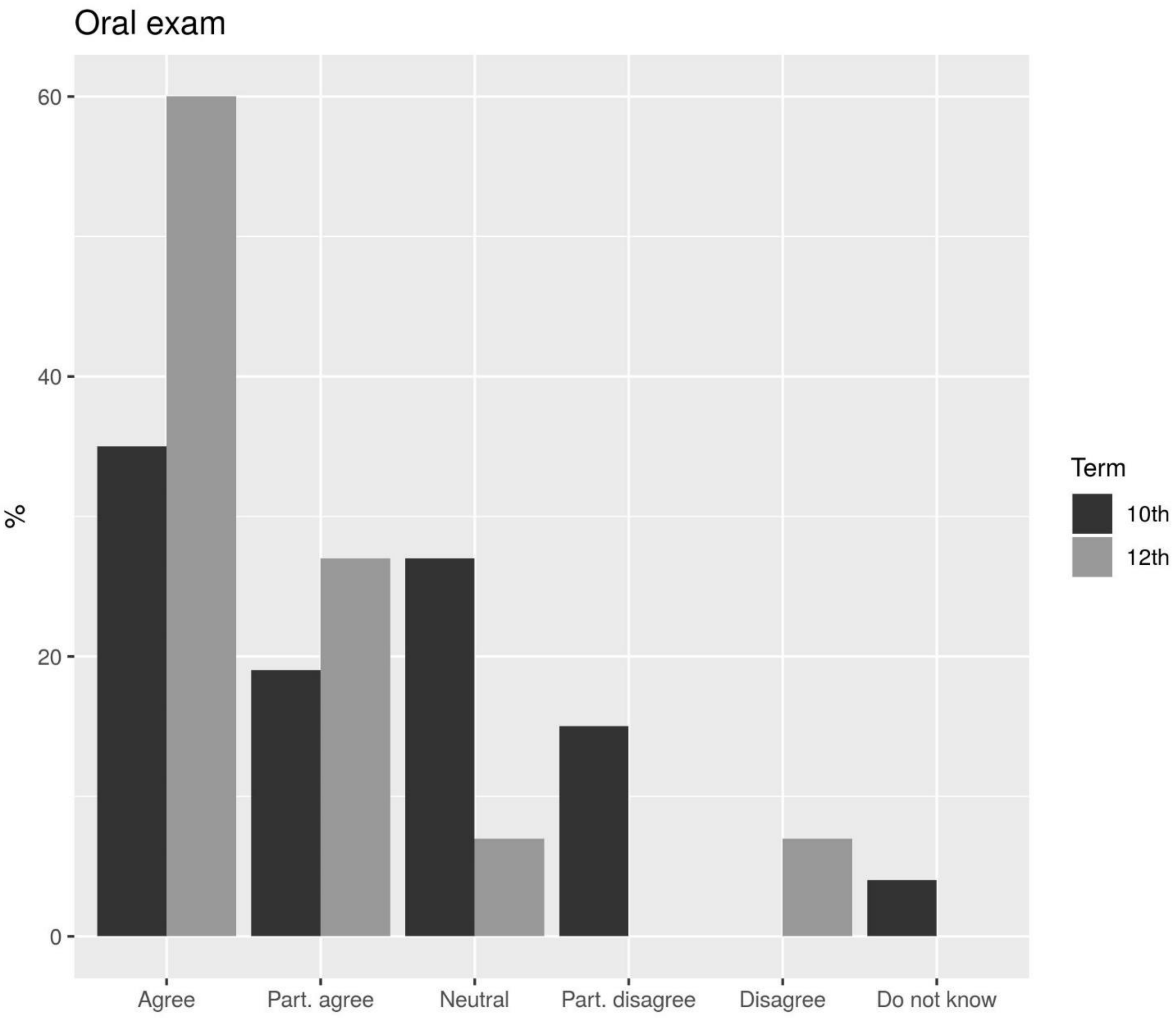

Authors' comment: The radiology course (MEDRAD) under the old curriculum (12th term) concludes with a multiple-choice examination in nuclear medicine and a 30-minute oral examination in radiology. There are no oral examinations under the new curriculum (10th term). It is noteworthy that the students who are exposed to oral examinations answered more in the negative than those who were not (n.s.).

Question 12: Multiple-choice examination

"Multiple-choice examinations on vurdering.uib.no/Inspera promote to my learning."

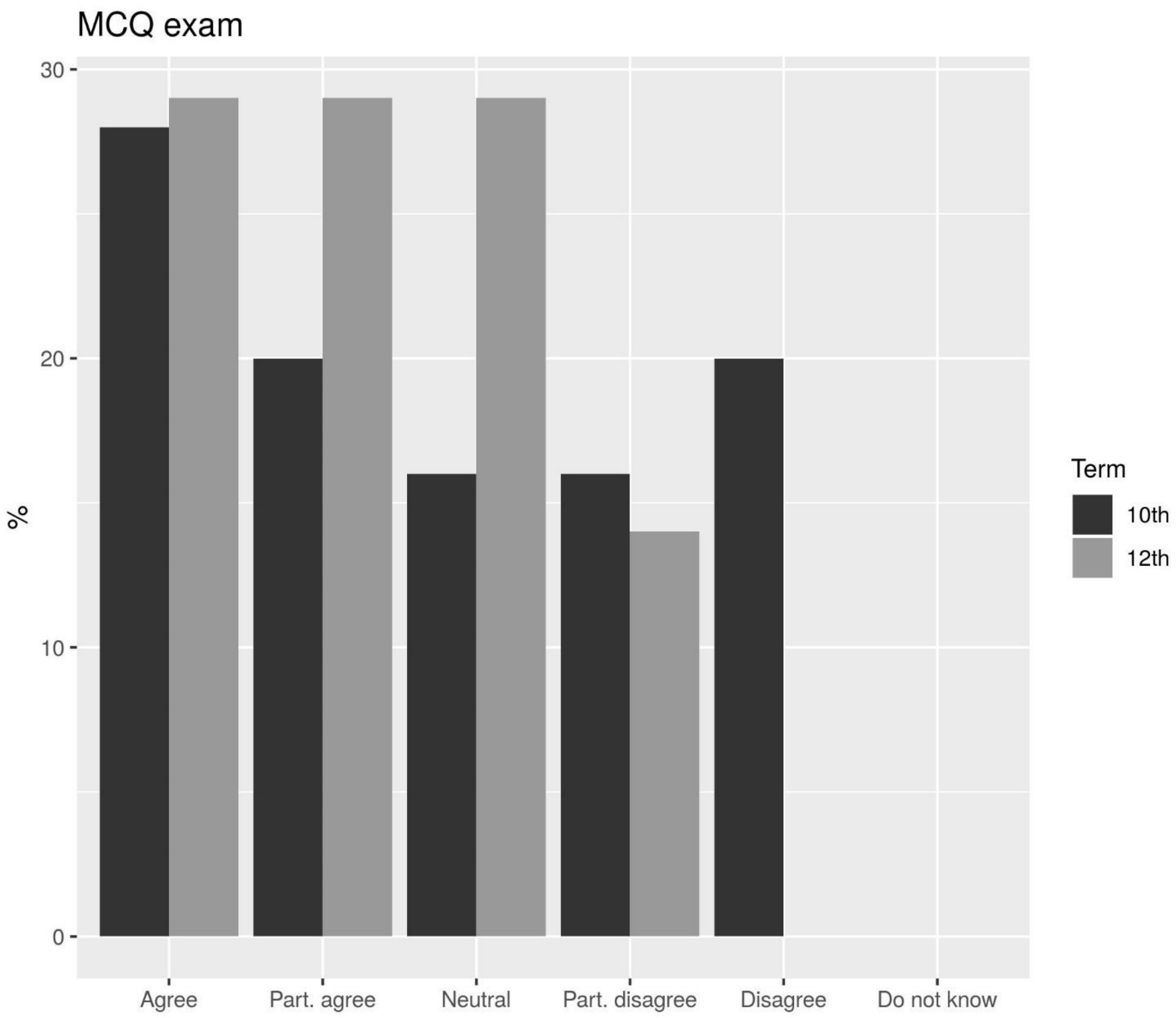

Authors' comment: The radiology course (MEDRAD) under the old curriculum (12th term) concludes with a multiple-choice examination in nuclear medicine. This examination is performed on a different portal (Inspera; https://vurdering.uib.no) than the system used for e-learning (Canvas LMS; https://mitt.uib.no). Under the new curriculum (10th term), students undergo an MCQ examination at the end of each term (except the 10th). It is interesting to note that the students who were most exposed to MCQ answered more in the negative (compare also question 11) (n.s.).

Question 13: Written examination (free-text)

"Written examinations with free text answers promote my learning."

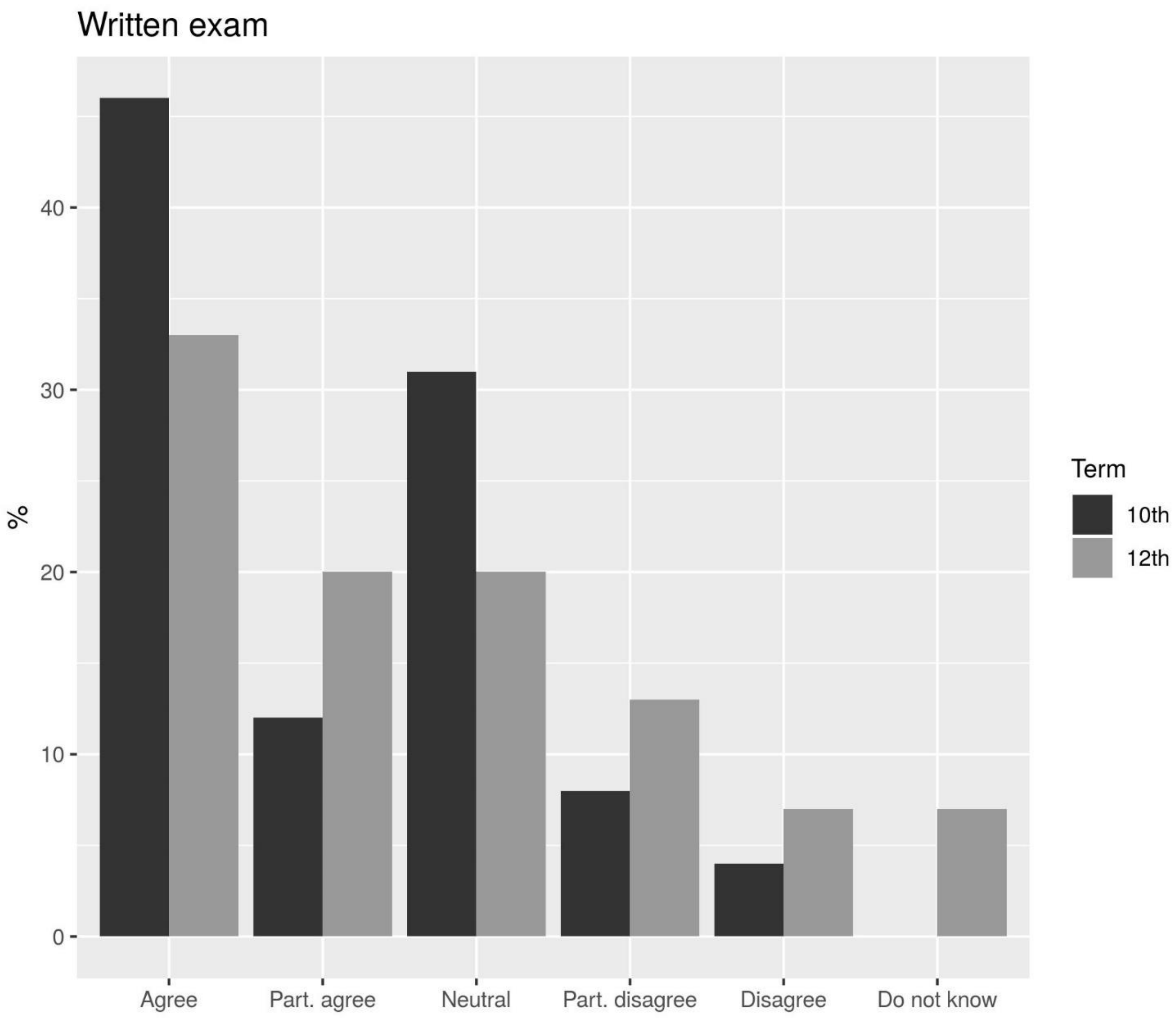

Question 14: Practical examination (OSCE)

"Practical examinations (OSCE) promote my learning."

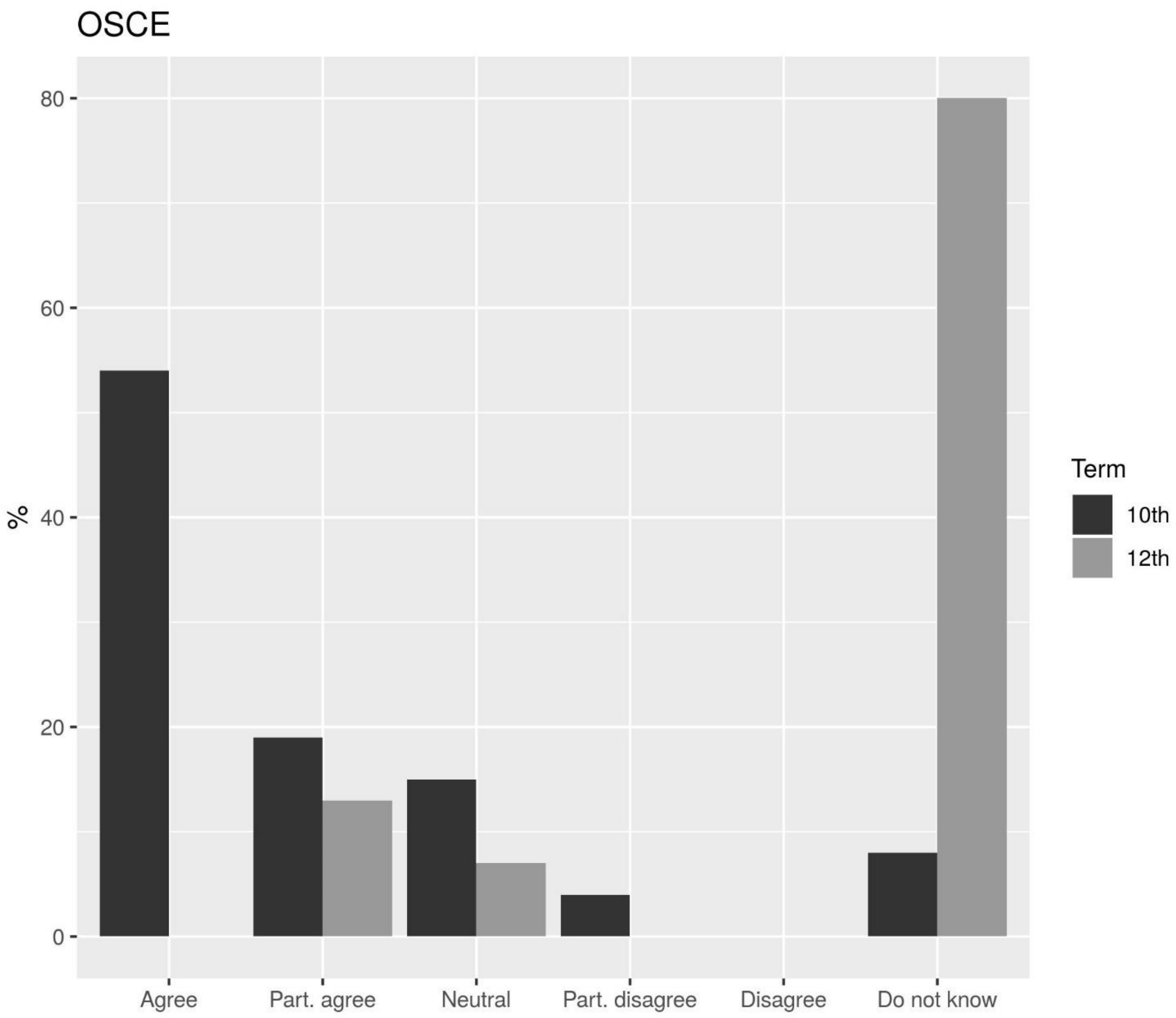

Authors' comment: The University of Bergen launched an Objective Structure Clinical Examination (OSCE) for 6th term medical students for the first time in June 2018. Nuclear Medicine contributed with one station. Some students in the 10th term had previously taken part in a voluntary OSCE pilot, but none of the students in the 12th term. 10th term students will undergo OSCE as part of their final examination in 2020.

Questions 15 to 17: Free-text questions: MORE, LESS, Comment

The last 3 questions were answered in free text format: "What would you like to have MORE of in teaching ("more")? What would you like to have LESS of in teaching ("less")? Do you have any further comments ("Comment")?"

To enhance readability, the responses of each respondent are listed sequentially according to student term. Respondents are counted sequentially within each student term. Occasional explanatory comments by the authors are enclosed in square brackets [].

10th term students (n = 26)

Eighteen of the 26 respondents (69 %) in the 10th term answered at least one of the three free text questions.

Student 3/26

More: Very much liked the web lecture which explained how to use Oasis. I would've liked to learn the rest of radiology in the same way!

Comment: Very well-designed MCQ, Martin. One gets feedback why something is wrong. Many questions that make sure that you cannot cheat your way through.

Authors' comment: The student's comment refers to the multiple-choice questions in the teaching material on the e-learning system mitt.uib.no; there was no multiple-choice examination in the 10th term.

Student 4/26

More: It is very exciting with exercises in which one really tries out Oasis *[the image viewing software]*. I think five compulsory teaching cases are enough, but it would be nice if we had access to some more, so we could try if we wanted to.

It would have been nice with an extra minute or two in the web lecture about how one decides on the treatment based on the imaging findings. When is surgery no longer advisable, when external beam radiation? I understand that this mainly will be taught within oncology, but a short summary would have been super.

It is very nice with web lectures so that one knows quite concretely what one needs to do in order to prepare for the seminar.

Less: not sure.

Comment: I constantly and on all occasions praise how good the teaching in nuclear medicine is. Thanks a lot that you talk yourself the time to make such a good course for us.

Authors' comment: We see that the web lecture needs a minor revision. The main decision in treating newly diagnosed lung cancer is curative versus palliative treatment. When going for curative therapy, the decision in favor of surgery versus stereotactic therapy is largely based on the patients presumed remaining lung function after curative surgery. When the predicted lung function is too poor, patients receive radiotherapy rather than surgery.

Student 5/26:

Comment: Teaching in nuclear medicine in this semester has been incredibly good. Short, good web lectures, concrete information on how we can prepare ourselves the seminar and incredibly instructive compulsory exercises in Oasis! The latter we could very well have much more of in radiology – e.g. diagnose fractures and the like. We would learn much in a fun and clinically relevant manner! I would recommend that you give clear advice that the compulsory exercises can be done in small groups of 2 to 4 students as there are not many computers with the software installed.

Student 7/26:

More: More practice! The most rewarding was to do the compulsory exercises after the seminar in Oasis to understand how the imaging software works.

Less: I think this was a very well-designed and good course. I think that all the subject matter is somewhat stressing and difficult to keep in mind, but I understand that we need this in order to understand the examinations performed in the patients.

Comment: I think this was a fantastic course, having a web lecture with the basal facts in the beginning and some tasks in the e-learning system (mitt.uib.no) where I got immediate feedback. I also learned a lot by working with the subject matter on my own. I would wish that all radiology teaching was designed in this way since I feel that we are a student term who has not got proper systematic radiology teaching. If we could have similar teaching in x-ray, CT and MR of different important organs (skeleton, abdomen, thorax, brain), this would've been very useful for our future clinical work. This is what I really wish before I complete my studies!!!

Student 9/26:

Comment: Absolutely fantastic teaching – if we had this in all subjects we would learn very much! I especially liked the web lectures so that we could prepare us (and look at it again if there was something that we had missed), that they were good to describe what were normal findings and pathology, and Oasis *[the medical image viewing software]* with feedback to the compulsory exercises (on mitt.uib.no) so that we could learn continuously. I would wish all radiology teaching was like this.

Student 10/26:

More: I like the concept that you challenge us students to sit down in front of a computer and find pathologic findings etc.

Less: Continuation from the previous question: if anything should be done, there should be more progress for the student, otherwise it gets somewhat tedious (especially if teaching is on the late afternoon).

Comment: Very cool to work with the lung cases in Oasis. This was the closest I ever came to navigating in a computer program related to imaging (I have not access to other programs under hospitalizations in peripheral hospitals). Continue with this, please make similar exercises for other areas. This is the closest many of our students will come to practical use of PET/CT before we become physicians.

Authors' comment: The "Less" comment apparently refers to the seminar. It is sometimes challenging to pace case presentations correctly so that no student is bored but every one understands. We now let students present the cases under supervision. This tends to maintain student engagement at a higher level than when the teacher keeps running the entire seminar. Most seminars took place in the morning, however one group had the seminar from 2:15 p.m to 4 p.m.

Student 13/26:

More: More about when one refers to PET, scintigraphy+++ I have no clue when one should or ought to do this.

Less: Less teaching in the claustrophobic room H113 (or so) down below. Much better to look at videos at home when it suits me.

Comment: Fantastic videos, but I would like more practical information when I should send patients to these special examinations.

Authors' comment: We deliberately do not focus on indications for nuclear medicine examinations. Indications for imaging examinations are an endless field that requires deep knowledge of the affected specialties including alternative diagnostic approaches such as clinical chemistry or biopsies that cannot be assumed in a normal 3rd or 5th year student. Teaching indications would take away the focus from the images and the key image findings. In the context of practical medicine, not teaching indications does not pose a problem, since all modern diagnostic/therapeutic algorithms will contain concrete advice on which imaging modality to choose in a given clinical situation.

Student 15/26:

More: More cases, even though there are many already.

Authors' comment: We are planning to create more cases in the context of future courses such as ELMED318 in January 2018. The teaching material in these courses will also be available made to regular students.

Student 16/26:

More: More tests on mitt.uib.no so that one could test oneself after the teaching. This has been very good in the 10th term :)

I often think that teaching can be very theoretical, so therefore [I wish] more concrete and practical in respect my future work as a physician.

Student 17/26:

Comment: There are too few computers with access to Oasis *[the medical image viewing software]*. We were not allowed to be in the clinical training center of the hospital because they often had courses ongoing, and we had no access to teaching room H 113. Therefore, we could only use the two computers at Armauer Hansen's house.

Authors' comment: For the time being, there are indeed too few computers that give medical students access to teaching resources in the hospital network including the medical image viewing software Oasis. This should be taken up when the University and the University Hospital convene to allocate resources for teaching medical students. (The University of Bergen and Haukeland University Hospital have separate networks, and all patient-related information including the teaching case database is available in the hospital network only.)

Student 18/26:

More: I could have wished to get more teaching on how one systematically can read images, so one is more confident on what is normal and what is pathological, and in order to see the less obvious pathological findings.

Student 20/26:

More: more opportunities to try out knowledge in practice. Very good exercises, multiple-choice on mitt.uib.no, cases etc.

Student 21/26:

More: More use of actual radiological software in order to learn more on how one uses CT and x-ray in practice. Group work. Net lectures with exercises afterwards.

Student 22/26:

More: Exercise-based learning together with other students or alone.

Less: Normal lectures.

Comment: I think that the course in nuclear medicine with web lectures and exercises has worked well.

Student 23/26:

More: I very much liked to work with Oasis *[the medical image viewing software]* and patient cases. This gave me the most!

Comment: I like the teaching setup by Biermann. He is engaged, prepared, creative in his teaching methodology, and helpful.

Student 24/26:

More: Practical exercises.

Less: Plenary teaching.

Student 25/26:

More: Cases. Clinical relevance.

12th term students

Fourteen out of 15 12th-year students (93 %; except student #5) responded to at least one of the free text questions.

Student 1/15:

Comment: I would like to say that you did a fantastic job! You make nuclear medicine fun, and you make it clear that you want us to learn the field. You have put down a lot of time to produce teaching material for preparing the MCQ exam for us; more lecturers should put their lectures on the Internet the way you do. Continue on these lines, is my advice. Thank you.

Student 2/15:

More: More clinical in respect what is important to know in clinical practice. I feel that we learned many things that I got the impression are not used much in clinical practice, for example thyroid scintigraphy (according to endocrine surgeons).

Comment: I very much liked the way the teaching was conducted. Unfortunately, I did not realize that we should prepare us for the seminar with web lectures before day 1 since we have so many different subjects and groups to relate to in the 12th term. The setup was very good and very helpful for preparing the examination. Interactive and good teaching!

Authors' comment: The first remark of the student points out a deficiency with the old curriculum – subjects were taught in parallel without much coordination. Thyroid scintigraphy is not so important in the context of thyroid surgery but much in demand by endocrinologists.

Student 3/15:

Comment: Thank you! What a well-organized subject. The web lectures are absolute gold – phantastic for preparing the seminar and as a repetition. In addition, very nice to have so many *[MCQ]* questions at our disposal on mitt.uib.no to train for the examination!

Student 4/15:

More: The type of web lectures that you already have in both nuclear medicine and radiology is worth gold. For my own sake, one could almost have dropped the two weeks with radiology lectures and rather have done something else since the material also was posted on the UiB website as prerecorded lectures which went through EXACTLY the same, but much more effectively and better. (However I got no information that these lectures were posted on the Internet through the lecturers or the faculty, but rather by chance through a fellow student – this could have been said more clearly, but then maybe fewer student would have shown up…)

Less: Plenary lectures where one just reads from a PowerPoint presentation and goes through images which one could do equally well on one's own when one wanted/was not tired.

When one should have presentations of cases in PowerPoint (especially in a subject like radiology) in the first place, it is hopeless when the PowerPoint files that are posted after the seminar with these cases do not contain explanations in PowerPoint of what findings these images actually have – with text and arrows on the images in order to explain. I understand that you want to have "incentives" so that people should meet at the lectures. But if the lectures are not good enough on their own so that people meet up, you should not "punish" those who do not show up because you have not sufficiently good lectures…

Comment: As I said, I praise the web lectures!

Nuclear medicine could maybe at times come down and seen which subject matter it focused on. You teach residents, not nuclear physicians. Use more time on what a resident actually may need.

Authors' comment: The student's criticism on lecture handouts mostly refers to radiology teaching in general. In our nuclear medicine teaching we have tried to reduce lecturing in face-to-face teaching to a minimum and use web lectures instead. All lecture handouts in nuclear medicine do contain clear labelling of the pertinent image findings. We concede the last criticism, under the new curriculum we have focused on what is needed by the average resident rather than teaching nuclear medicine as such.

Student 6/15:

More: Even more cases.

Comment: I think it is incredibly good that you made web lectures, with soundtrack and PowerPoint! They are incredibly instructive, and one can look at them when one self wants to, and that they are relatively short and concise. The result is that one actually manages pay attention.

I am very happy about nuclear medicine, this could have been a rather peripheral and boring subject, but the way it has been structured with quizzes and net-based case exercises has made this very good!

Student 7/15:

Comment: thank you very much for all the work you have invested in the course, this is the best I have experienced in the whole of my medical studies. Unfortunately, the specialty is in the shadow of internal medicine and surgery, just as earlier teaching in radiology came in the shadow of other examinations. I would have rather had the whole specialty in one unit in the 4th or 5th year, then the teaching outcome would have certainly been even greater.

Student 8/15:

More: Group exercises? Probably more use for future students undergoing OSCE.

Comment: Biermann is probably the lecturer at the medical faculty of the University of Bergen who has spent most effort on a teaching setup that is well-defined, up-to-date, accessible and "multimodal" utilizing several teaching modes. Teaching and learning outcomes are largely in alignment with clinical relevance for a "general practitioner", which is what we are actually being trained for. Other subjects, hark, hark!

Student 9/15:

Less: Focus on particular isotopes for different examinations. As I see it, it is more relevant to learn the principles behind nuclear medicine, which applications they have, and when one potentially can have benefit of such an examination. [This is more important] than to know that it is ^{99m}Tc-MIBI one uses for cardiac scintigraphy and parathyroid scintigraphy, because it difficult for me to see that this is something one must specify when one should refer a patient to a nuclear medicine examination sometime in the future?

Comment: I hope that radiology will be moved to an earlier part of the new curriculum. This is an incredibly useful subject that one should rather learn before the first placement at a peripheral hospital in order to increase learning outcome. When the radiology exam comes so close to the final exams, the effect is that one tries to use as little time as possible on radiology because internal medicine and surgery are looming and so close. Which is very regrettable! Otherwise I want to thank you all for very good teaching in radiology, good lecturers who also manage to differentiate between what we should know as residents and what is more advanced specialist knowledge.

Authors' comment: We agree - the student should know the basic type of nuclear medicine examination and the principles behind it does not need to memorize the name of the radioactive drug. However, there are many different radioactive drugs that can be used for a given nuclear medicine examination so we always have to indicate which precise drug was used. For clinical referrals, it is paramount that the physician describes the clinical scenario and the clinical question, and we will choose the most appropriate study regardless which tracer was suggested in the referral. – In the new curriculum, teaching of radiology and nuclear medicine is no longer concentrated in the last 3 months before the final examination.

Student 10/15:

More: Very good with quizzes and Socrative [https://socrative.com] in teaching. Continue with this and do more of this instead of lectures. Continue to ask students to prepare themselves before showing up.

Less: Fewer pure lectures.

Comment: Very good course design.

Student 11/15:

More: Training in reading images.

Less: Happy [sic!].

Student 12/15:

More: More cahoot.it/Socrative. More MCQ exercises on mitt.uib.no

Less: Fewer plenary lectures.

Student 13/15:

More: I think that the teaching a nuclear medicine has been very good, and I feel that I have good knowledge on the most important applications and I know when I shall refer.

Less: Details about names of different substances that are injected is knowledge that is soon forgotten, and I'm not sure how much focus one should put on this.

Comment: Thanks a lot for the effort that your put in to make web lectures, quizzes etc. This has been very good!

Student 14/15:

More: A larger number of relevant exercises on mitt.uib.no with images. This is a very visual specialty in which one needs to look at and read images. The web lectures were also very good and informative.

Less: Less "whiteboard/PowerPoint teaching "

Authors' comment: We heartily agree with you. In the most recent redesign of the course PowerPoint is used for the web lectures only, and Face-to-face teaching is largely based on the interactive presentation of cases using dedicated nuclear medicine software such as Oasis. We resort to Powerpoint only when specific questions come from the audience that are best explained using a PowerPoint slide.

Student 15/15:

Comment: I remember that I was happier with the 1st day of nuclear medicine than with the other day. But good teaching on both days.

Authors' comment: The 1st day of teaching was on organ diagnostics, the 2nd day was on oncology.
